# Extension of Indication for Authorised Oncology Products in the European Union: A Joint Effort of Multiple Stakeholders

**DOI:** 10.3389/fmed.2021.790782

**Published:** 2021-12-09

**Authors:** Jorn Mulder, Robin Verjans, Ciska Verbaanderd, Elias Pean, Just Weemers, Hubert G. M. Leufkens, Francesco Pignatti, Anthonius de Boer, Emile E. Voest, Violeta V. Stoyanova-Beninska, Anna M. G. Pasmooij

**Affiliations:** ^1^Dutch Medicines Evaluation Board, Utrecht, Netherlands; ^2^Lygature, Utrecht, Netherlands; ^3^European Medicines Agency, Amsterdam, Netherlands; ^4^Department of Pharmaceutical and Pharmacological Sciences, KU Leuven, Leuven, Belgium; ^5^Janssen Biologics BV, Leiden, Netherlands; ^6^Utrecht Institute for Pharmaceutical Sciences, Utrecht University, Utrecht, Netherlands; ^7^Department of Molecular Oncology and Immunology, The Netherlands Cancer Institute, Amsterdam, Netherlands; ^8^Oncode Institute, Amsterdam, Netherlands

**Keywords:** investigator-initiated trials, extension of therapeutic indication, regulatory approval, European Medicines Agency, anti-cancer medicinal products

## Abstract

After marketing authorisation, the development of a medicinal product often continues with studies investigating new therapeutic indications. Positive results can potentially lead to changes to the terms of the marketing authorisation, such as an extension of therapeutic indication(s). These studies can be initiated and sponsored by the marketing authorisation holder (MAH) or by others. When results from an investigator-initiated trial suggest that an authorised medicinal product is safe and effective for a new therapeutic indication, physicians may want to treat their patients with this medicinal product. In such a situation, it is desirable to extend the therapeutic indication(s) *via* the regulatory approval process, as this can facilitate patient access within the European Union. There may however be challenges when the MAH did not conduct the study and might not have access to the data. In this perspective, we focus on the possibilities to extend the therapeutic indication(s) of an already authorised medicinal product based on results from investigator-initiated trials. We address: (1) the advantages of an extension of indication; (2) the regulatory requirements for a variation application; (3) investigator-initiated trials as a basis for regulatory approval; (4) the role of the MAH in extending the indication. With this article, we want to emphasize the importance of a collaborative approach and dialogue between stakeholders with the aim to facilitate access to effective medicinal products.

## Introduction

After marketing authorisation, the development of a medicinal product often continues with studies investigating new therapeutic indications. Positive results can potentially lead to changes to the terms of the marketing authorisation, such as an extension of therapeutic indication(s). Studies investigating new therapeutic indications can be initiated and sponsored by the marketing authorisation holder (MAH) or by others, such as academic researchers. Studies initiated by academic researchers are referred to as “investigator-initiated studies,” and can be conducted independently or via different forms of collaboration with the MAH. There are several examples of investigator-initiated studies in the area of oncology, including the Drug Rediscovery Protocol (DRUP).

The DRUP is an ongoing, national, prospective, multi-drug and pan-cancer trial sponsored by the Netherlands Cancer Institute (ClinicalTrials.gov Identifier: NCT02925234; EudraCT Number: 2015-004398-33) (1). In the DRUP, 35 anti-cancer medicinal products, including those still on-patent, are used outside of the terms of their marketing authorisation to treat treatment-exhausted patients with metastatic cancer that harbour an actionable oncogenic driver (1). van der Velden et al. reported the study design and first treatment results in 2019 (1); in short, a two-stage design was used for each cohort. As per protocol, cohorts consisting of a tumour type, a molecular target and a matched treatment were considered successful if ≥5 out of 24 patients had either complete or partial response, or absence of disease progression for ≥16 weeks (1). Recently, Hoes et al. presented the results of the first 500 patients, and showed that the cohort of patients with microsatellite instable (MSI) tumours treated with nivolumab, and the cohort of patients with BRCA-positive tumours treated with olaparib were considered successful (2).

Nivolumab and olaparib are authorised in the European Union (EU), but not for the treatment of MSI tumours or for the treatment of BRCA-positive tumours, respectively, i.e., so-called tissue-agnostic indications. A third stage is added to the DRUP that allows for partial reimbursement as well as confirmation of the results observed in the earlier stages of the trial (3). The nivolumab cohort already expanded to this stage, and similar plans for olaparib are in an advanced phase. This performance-based, personalised reimbursement scheme is currently running as a pilot in the Netherlands (3). Yet, in other EU member states, the unauthorised use of these medicinal products might not be reimbursed.

When results from an investigator-initiated trial suggest that an authorised medicinal product is safe and effective for new therapeutic indications, physicians may want to treat their patients with this medicinal product. In such a situation, it is desirable to apply for an extension of the therapeutic indication(s) via the regulatory approval process, as this can facilitate patient access within the EU. To initiate this process for (anti-cancer) medicinal products authorised via the centralised procedure, the MAH needs to submit a variation application to the European Medicines Agency (EMA). There may however be challenges when the MAH did not conduct the study and might not have access to the data. Here, the DRUP is used as an example of an investigator-initiated trial, but it should be noted that the adequacy of the dataset to support an extension of indication has not formally been assessed by regulatory agencies.

On 23 June 2020, a Regulatory Science Network Netherlands (RSNN) expert meeting that focussed on “Label modification based on evidence deriving from investigator-initiated trials” was held (4). During this meeting, the DRUP was used as an example and the need to extend the therapeutic indication(s) based on results from investigator-initiated trials, ownership of data, and regulatory possibilities were discussed. Here, we want to elaborate on the latter, as during the expert meeting it became clear that more information on this topic is warranted. Therefore, we consider it of relevance to further discuss the possibilities concerning the addition of a new therapeutic indication to an already authorised medicinal product based on results from investigator-initiated trials. This will become increasingly important as the growing experiences with precision medicine, advancements in technology and use of innovative trial designs (e.g., basket and umbrella trials) contribute more efficient development of medicinal products, especially in the field of oncology. We specifically focus on medicinal products that are still on-patent and are approved via the centralised procedure, but many aspects discussed below also apply to off-patent medicinal products. This article is a collaborative approach from authors with different affiliations, since this topic concerns several stakeholders.

## Advantages of an Extension of the Therapeutic Indication

Reimbursement of off-label use depends on national health insurance legislation. In most EU member states, reimbursement is limited to approved therapeutic indication(s) (5). Hence, when the benefit-risk balance could be considered positive, an extension of the therapeutic indication(s) is warranted. Importantly, an application for the addition of a new therapeutic indication triggers an independent assessment of the efficacy and safety data that are submitted. A new therapeutic indication will be approved only if the benefit-risk balance is considered positive by regulators. In addition, the benefit-risk balance is re-evaluated on a continued basis taking into account potential new safety findings in the post-marketing setting (6). Besides, liability issues for prescribers can arise if a medicinal product causes adverse reactions when used off-label, which can be prevented by regulatory approval (5).

## Regulatory Requirements for a Variation Application

To extend the therapeutic indication(s) of a medicinal product approved via the centralised procedure, the MAH has to submit a type II variation application to the EMA (7). A variation application concerning the addition of a new therapeutic indication shall comply to the same standard data requirements as for an initial marketing authorisation application (MAA) with regards to the evidence required to demonstrate safety and efficacy. Clinical standards and protocols in respect to the testing of medicinal products are described in detail in Annex I of the Directive 2001/83/EC (8). With regulatory purposes in mind, data requirements would apply to any clinical trial, regardless of its sponsor.

The type of evidence necessary to demonstrate the efficacy and safety of a medicinal product are defined by EU law (9). However, the amount of evidence that can be gathered will not always be similar. For instance, the rarity of a disease, or even the incidence of an actionable oncogenic driver, may impact the feasibility of conducting large randomised controlled trials (RCTs). This has also been addressed in the EMA Committee for Medicinal Products for Human Use (CHMP) draught guideline on the clinical evaluation of anticancer medicinal products, which includes a section on specific designs for specific situations (10). While RCTs are still considered the gold standard for the demonstration of efficacy and safety in a new therapeutic indication, there are examples where results from trials with alternative designs have supported a variation. For example, the extension of indication for crizotinib to include treatment of adult patients with ROS1-positive advanced non-small cell lung cancer (NSCLC) was supported by results from a single-arm trial, considering the high response rate observed and that ROS1-positive NSCLC represents a rare, serious and life-threatening distinct molecular subset (11). The scientific evaluation of a variation application is done on a case-by-case basis, taking into account all relevant factors, including those mentioned above. Before submitting a variation application, the MAH could consider to request scientific advice from regulatory authorities to discuss the use of results from an investigator-initiated trial to support the extension of indication.

## Investigator-Initiated Trials as a Basis for Regulatory Approval

The MAH does not have to be the sponsor of the clinical trial to apply for an extension of indication, as long as he has access to the data. For example, an extension of indication for rituximab for the treatment of adult patients with pemphigus vulgaris was supported by results from an investigator-initiated trial, and the sponsor of the clinical trial transferred all necessary data to the MAH before submission (12). Alternatively, if the MAH does not have access to the data, bibliographic references can be used to support a variation application. The pharmaceutical legislation allows for mixed marketing authorisation applications dossiers where parts of modules 4 (non-clinical reports) and/or 5 (clinical study reports) are replaced by bibliographical references (9). An example is the extension of the indication for arsenic trioxide in combination with all trans-retinoic acid for first-line treatment of acute promyelocytic leukaemia (13). In this variation, results were submitted in the form of bibliographic references, but it is noteworthy that the data included in these references were considered sufficiently detailed – allowing for a thorough scientific evaluation.

Stakeholders other than the MAH cannot submit a variation application concerning the addition of a new therapeutic indication, since they are not the owner of the marketing authorisation. The possibilities to evaluate data from investigator-initiated trials by European regulators without the involvement of the MAH have been discussed during several meetings of the Commission Expert Group on Safe and Timely Access to Medicines for Patients (STAMP) and during an *ad hoc* session with stakeholders in the context of the development of a framework for the repurposing of established medicines (14). An opinion on a scientific matter can be drawn up by the EMA/CHMP at the request of the Executive Director of the Agency or the Commission representative without the direct involvement of the MAH(s), namely via an Article 5(3) procedure of Regulation (EC) No 726/2004 (15). However, this is an exceptional procedure in emergency situations or where there is a high public health interest on a focused scientific issue. In September 2020, the EMA endorsed the use of dexamethasone in hospitalised patients with COVID-19 based on the results from the investigator-initiated RECOVERY trial, following an Article 5(3) procedure triggered by the Executive Director of the EMA (16, 17). The EMA published that the new use for dexamethasone can be added to the product licence upon request by a MAH (17). Yet, following an Article 5(3) procedure, the MAH(s) would still need to submit a variation application before any changes to the terms of the marketing authorisation can be made, but the MAH is not obligated to do this.

## The Role of the MAH in Extending the Therapeutic Indication

As described by Rauh et al., the MAH remains a central player when considering an extension of indication (18). Addressing the various reasons why the MAH may, or may not, want to apply for an extension of indication is outside the scope of this article, but a few reasons that might influence the preparedness of the MAH to apply for an extension of indication are discussed below. The MAH would need to prepare and submit an application, which costs time and resources, while the outcome of the assessment is uncertain. It should be noted that specific regulatory exclusivities exist in Europe to incentivize companies to invest in the development of new indications for authorised products (19). However, several criteria need to be met for a product to be eligible for such incentives and previous research has shown that the available incentives may not be enough to stimulate the development of new indications (20, 21). Also, the MAH may prioritise the development of other products included in its pipeline or might simply not be interested in extending the therapeutic indication(s) because the new indication is outside their therapeutic focus. In some EU countries, the pricing of the medicinal product will be re-negotiated after a new therapeutic indication is added to the terms of the marketing authorization (19). There is a risk that the price of a medicinal product decreases following the extension of indication (22), which may represent a barrier for MAHs when considering the addition of a new therapeutic indication.

## Discussion

When results from well-conducted investigator-initiated trials establish that an authorised medicinal product can be used outside the terms of the marketing authorisation, patients should be given the opportunity to be treated with such a medicinal product. Extending the therapeutic indication(s) would allow an independent assessment of the benefit-risk balance of a medicinal product in that specific indication and approval may facilitate reimbursement. In addition, extending the therapeutic indication(s) would decrease the gap between clinical practise and regulatory approval.

It is important to discuss among stakeholders the regulatory possibilities in case (robust) evidence on the use of a medicinal product outside the therapeutic indication(s) emerges from investigator-initiated trials, especially if there is an unmet medical need. The MAHs should not be reluctant to use results from investigator-initiated trials to support an extension of indication, as long as standard regulatory requirements are met. Therefore, early dialogue between regulators and the MAH to discuss the proposed indication and the use of results from investigator-initiated trials can be helpful, for instance via scientific advice. In addition, the importance of scientific advice was highlighted by the Commission Expert group STAMP as a way to support academic researchers in designing pivotal clinical trials that meet regulatory standards and generate comprehensive data in the context of repurposing established medicines (23), which is of importance if the trial has not yet been initiated. It is essential to ensure that investigator-initiated trials meet the standard quality requirements such as good clinical practice, especially if these trials will be used for regulatory purposes. In the context of future revision of the pharmaceutical legislation, there is a need to consider a mechanism to evaluate results from investigator-initiated trials without the involvement of the MAH. This may stimulate MAHs to submit a variation application after a positive opinion has been issued at EU level.

In conclusion, it is possible to support an extension of indication by results from investigator-initiated trials, but regulatory requirements still need to be met. We want to emphasise the importance of a collaborative approach and dialogue between stakeholders with the aim to facilitate access to effective medicinal products. In the end, the data tell the story and should make the difference.

## Data Availability Statement

The original contributions presented in the study are included in the article/supplementary material, further inquiries can be directed to the corresponding author/s.

## Author Contributions

All authors listed have made a substantial, direct, and intellectual contribution to the work and approved it for publication.

## Author Disclaimer

The views expressed in this comment are the personal views of the author(s) and may not be understood nor quoted as being made on behalf of, or reflecting the position of, their organisations.

## Conflict of Interest

EV initiated and led the DRUP as one of the principal investigators. The remaining authors declare that the research was conducted in the absence of any commercial or financial relationships that could be construed as a potential conflict of interest.

## Publisher's Note

All claims expressed in this article are solely those of the authors and do not necessarily represent those of their affiliated organizations, or those of the publisher, the editors and the reviewers. Any product that may be evaluated in this article, or claim that may be made by its manufacturer, is not guaranteed or endorsed by the publisher.
